# Presenting Twins Are Exposed to Higher Levels of Inflammatory Mediators than Nonpresenting Twins as Early as the Midtrimester of Pregnancy

**DOI:** 10.1371/journal.pone.0125346

**Published:** 2015-06-15

**Authors:** Seung Mi Lee, Joong Shin Park, Errol R. Norwitz, Sun Min Kim, JoonHo Lee, Chan-Wook Park, Byoung Jae Kim, Jong Kwan Jun

**Affiliations:** 1 Department of Obstetrics & Gynecology, Seoul National University College of Medicine, Seoul, Korea; 2 Department of Obstetrics & Gynecology, Seoul Metropolitan Government Seoul National University Boramae Medical Center, Seoul, Korea; 3 Department of Obstetrics & Gynecology, Tufts University School of Medicine, Boston, Massachusetts, United States of America; CHA University, REPUBLIC OF KOREA

## Abstract

**Objective:**

Presenting twins are less likely to develop respiratory complications than non-presenting twins. The precise reason for this difference is not well understood, although it is known that the presence of inflammation reduces the risk of respiratory morbidity at birth. To further investigate this association, we compared the concentrations of inflammatory biomarkers in mid-trimester amniotic fluid (AF) of asymptomatic twin pairs.

**Study Design:**

The study population consisted of women with twin pregnancies who underwent mid-trimester amniocentesis (15–20 weeks) for routine clinical indications and delivered at term. AF was analyzed for pro-inflammatory cytokines (IL-1β, IL-2, IL-4, IL-5, IL-6, IL-8, IL-10, IL-12, IL-13, IL-15, IFN-γ, TNF-α), matrix metalloproteinases (MMP-1, MMP-2, MMP-3, MMP-8, MMP-9, MMP-12), and chemokines (Complement Factor D/Adipsin, Serpin E1/PAI-1, Adiponectin/Acrp30, CRP, CCL2/MCP-1, Leptin, Resistin) using Luminex Performance Assay multiplex kits. Data were analyzed using Wilcoxon signed rank test.

**Results:**

A total of 82 twin pairs were enrolled. Mid-trimester AF concentrations of IL-8, MMP-8, CRP, MCP-1, leptin, and resistin were significantly higher in the presenting twin compared with the non-presenting twin (p<0.05 for each). Differences in AF concentrations of IL-8, MMP-8, and CRP persisted after adjustment for the fetal growth restriction at the time of birth and chorionicity.

**Conclusion:**

These data suggest that, as early as the mid-trimester, the presenting fetus in an otherwise uncomplicated twin pregnancy is exposed to higher levels of pro-inflammatory mediators (especially IL-8, MMP-8, and CRP) than its non-presenting co-twin. Whether this pro-inflammatory milieu reduces the risk of neonatal respiratory morbidity at birth or has other functional implications needs to be further evaluated.

## Introduction

The incidence of twin births has increased dramatically over the past few decades primarily because of infertility therapy, making up 3.21% of all births in 2009 in the United States[1]. Twin pregnancies are associated with increased risks of neonatal mortality and morbidity compared with singleton pregnancies,[2–4] and non-presenting twins (second twins) are at higher risk for adverse outcomes than presenting twins (first twins) [5,6]. Mode of delivery has been implicated as one of the major causes of the increased mortality and morbidity in second twins, because adverse outcomes are more common when planned vaginal delivery is attempted[5,7–10].

Second twins are also more likely to develop respiratory complications than first twins, independent of gestational age and mode of delivery[11–13]. The precise reason for this difference is not well understood, although it has been shown that the presence of inflammation reduces the risk of respiratory morbidity in singleton pregnancies[14]. We postulate that first twins are exposed to higher levels of inflammatory mediators than second twins, and that this may be true throughout pregnancy and not only at term when the cervix is effaced and dilated thereby allowing for ascending infection and inflammation. To further investigate this association, we compared the concentrations of inflammatory biomarkers in mid-trimester amniotic fluid (AF) collected from asymptomatic twin pairs.

## Materials and Methods

### Study design

In this retrospective cohort study, consecutive twin pregnant women who underwent clinically indicated mid-trimester amniocentesis (15–20 weeks of gestation) for fetal karyotyping and delivered at term were enrolled. Cases with subsequent preterm delivery, major fetal structural anomaly or aneuploidy, or fetal death in utero were excluded. Amniocentesis was performed after informed written consent and an aliquot of amniotic fluid from each twin was stored at -70°C until assayed after centrifugation at 2000 rpm. The Institutional Review Board at Seoul National University Hospital approved the study and the patients provided their written consent for the collection and use of clinical information and these samples for research purpose.

### Quantification of proteins

Stored AF samples were analyzed for cytokines, matrix metalloproteinases and chemokines. Cytokines including interleukin (IL)-1ß, IL-2, IL-4, IL-5, IL-6, IL-8, IL-10, IL-12, IL-13, IL-15, IFN-γ, and TNF-α were measured with Bio-Plex Pro assays (BIO-RAD Laboratories, Inc., Hercules, CA, USA). Matrix metalloproteinases (MMPs; MMP-1, MMP-2, MMP-3, MMP-8, MMP-9, MMP-12) and chemokines (Complement Factor D/Adipsin, Serpin E1/PAI-1, Adiponectin/Acrp30, C-Reactive Protein, CCL2/MCP-1, Leptin, Resistin) were measured with Luminex Performance Assay (R&D Systems, Inc., Minneapolis, MN, USA). In these multiplex assays, the antibodies are covalently coupled to microspheres with a unique fluorescent dye, thereby enabling the determination of concentrations of each analyte using a Bio-Plex 200 analyzer (BIO-RAD). For the purpose of analysis, values below the lower limit of detection for each analyte were recorded as the lower limit of quantification (LLOQ).

### Statistical methods

The clinical characteristics and analyte concentrations in AF were compared between the first and second twin. Continuous variables were compared using Wilcoxon signed rank test and categorical variables were compared using McNemar test. A generalized estimating equation (GEE) was used to adjust confounding variables in the relationship between birth order and the AF concentrations of analytes. GEE has been used in multivariate analysis for multiple outcomes from the same subject[15,16] and in family-based association studies.[17] Statistical analyses were conducted using the IBM SPSS version 20. *P*<0.05 was considered significant.

## Results

During the study period, a total of 152 twin pregnant women underwent genetic amniocentesis at 15–20 weeks of gestation. Cases with major fetal anomalies (n = 7), cases which were lost to follow up (n = 19), and cases in which the presenting and non-presenting twins are not definitely defined (n = 3) were excluded. In the remaining 123 women, 82 women delivered at term and were included in the final analysis.

Table 1 shows the clinical characteristics of the study population. Amniocentesis was performed at a mean of 17.0 weeks of gestation, and the most common indication for genetic amniocentesis was advanced maternal age. The mean gestational age at delivery was 37.8 weeks and cesarean delivery was performed in 66% of cases.

**Table 1:** Clinical characteristics of the study population.

Characteristics	Twin pairs
	(n = 82)
Maternal age (years)	35 ± 3
Nulliparity	59 (72%)
Gestational age at amniocentesis (weeks)	17.0 ± 0.7
Indication for amniocentesis	
Advanced maternal age	68 (83%)
Abnormal serum screening	8 (10%)
Abnormal ultrasound	1 (1%)
Maternal request	5 (6%)
Gestational age at delivery (weeks)	37.8 ± 0.7
Birth weight (grams) *	2723 ± 337 (presenting twin) / 2667 ± 377 (non-presenting twin)
Cesarean delivery	54 (66%)

* p = NS between presenting and non-presenting twins (analyzed with Wilcoxon signed rank test).

Table 2 compares the concentrations of analytes in the AF collected from first and second twins. A total of 15 analytes were detected in significant quantities (i.e., were measurable in >90% of cases). Among these, the mean AF concentrations of IL-8, MMP-8, CRP, MCP-1, leptin, and resistin were higher in the first twin than in the second twin. (IL-8: 568.53 vs 448.16 pg/mL, respectively, p<0.05; MMP-8: 2090.55 vs 1680.63 pg/mL, respectively, p<0.05; CRP: 76.6 vs 49.3 ng/mL, respectively, p<0.05; MCP-1: 0.57 vs 0.52 ng/mL, respectively, p<0.05; Leptin: 17.0 vs 15.5 ng/mL, respectively, p<0.05; Resistin: 16.7 vs 14.6 ng/mL, respectively, p<0.01). The differences in AF concentrations of IL-8, MMP-8, and CRP between the first and second twins remained significant after adjustment for the fetal growth restriction at the time of birth and chorionicity (Table 2).

**Table 2:** Concentrations of analytes in mid-trimester amniotic fluid in presenting versus non-presenting twins.

Characteristics	unit	Presenting twin	Non-presenting twin	P value*	Percent <LLOQ
		(n = 82)	(n = 82)		
IL-1β	pg/mL	1.67 ± 1.23	1.66 ± 1.26	NS	100
IL-2	pg/mL	2.82 ± 2.62	3.25 ± 3.33	NS	84.1
IL-4	pg/mL	0.36 ± 0.24	0.36 ± 0.24	NS	98.2
IL-5	pg/mL	1.23 ± 1.54	1.23 ± 1.57	NS	100
IL-6	pg/mL	350.98 ± 542.31	279.52 ± 428.32	NS	1.2
**IL-8**	**pg/mL**	**568.53** ± **993.94**	**448.16** ± **654.53**	**<0.05** **†**	**0.0**
IL-10	pg/mL	3.45 ± 1.82	3.51 ± 1.69	NS	97.6
IL-12	pg/mL	3.43 ± 1.81	3.07 ± 1.66	0.091	99.4
IL-13	pg/mL	1.68 ± 1.63	1.67 ± 1.71	NS	100
IL-15	pg/mL	6.07 ± 7.12	6.17 ± 6.67	NS	60.4
GM_CSF	pg/mL	25.41 ± 8.36	24.61 ± 7.39	NS	0.0
IFN-r	pg/mL	7.61 ± 10.78	6.46 ± 8.37	NS	80.5
TNF-a	pg/mL	6.19 ± 2.94	6.26 ± 2.77	NS	100
MMP-1	pg/mL	760.96 ± 874.26	742.51 ± 855.49	NS	0.0
MMP-2	pg/mL	167922.2 ± 57259.1	167635.6 ± 63210.0	NS	0.0
MMP-3	pg/mL	1654.33 ± 910.69	1598.21 ± 956.81	NS	0.0
**MMP-8**	**pg/mL**	**2090.55 ± 2341.75**	**1680.63 ± 1991.43**	**<0.05** **†**	**1.2**
MMP-9	pg/mL	714.68 ± 584.30	673.85 ± 516.87	NS	7.3
MMP-12	pg/mL	21.76 ± 31.86	23.68 ± 38.30	NS	57.9
Complement factor D	ng/mL	1292.9 ± 510.3	1215.0 ± 603.9	NS	0.0
Serpin E1	ng/mL	177.7 ± 98.2	158.5 ± 100.1	0.052	0.0
Adiponectin	ng/mL	68.4 ± 35.6	61.1 ± 33.8	0.052	0.0
**CRP**	**ng/mL**	**76.6 ± 170.7**	**49.3 ± 79.5**	**<0.05** **†**	**0.0**
**CCL2/MCP-1**	**pg/mL**	**0.57 ± 0.26**	**0.52 ± 0.26**	**<0.05**	**0.0**
**Leptin**	**ng/mL**	**17.0 ± 11.0**	**15.5 ± 10.7**	**<0.05**	**0.0**
**Resistin**	**ng/mL**	**16.7 ± 11.8**	**14.6 ± 11.5**	**<0.01**	**0.0**

LLOQ, lower limit of quantification; NS, not significant.

* analyzed with Wilcoxon signed rank test.

† Significant after adjustment for fetal growth restriction and chorionicity.

## Discussion

### Main findings

The principal findings of this study were that AF concentrations of IL-8, MMP-8, CRP, MCP-1, leptin, and resistin were significantly higher in the presenting compared with the non-presenting twin already in the mid-trimester (p<0.05 for each). The differences in AF concentrations of IL-8, MMP-8, and CRP between the first and second twin remained significant after adjustment for the fetal growth restriction at the time of birth and chorionicity.

### Inflammation and respiratory morbidity in singleton pregnancy

Previous studies have shown that inflammation can promote fetal lung maturation in both animals and humans. In animal studies, intra-amniotic administration of endotoxin or inflammatory cytokine accelerated lung maturation by increasing the production of airway surfactant and improving lung gas exchange, which has been suggested as the mechanism by which antenatal corticosteroids act to promote functional maturation of the fetal lung[18–24]. Recent studies suggest that chorioamnionitis is associated with the decreased risk of neonatal respiratory distress syndrome, thereby supporting the theory that pulmonary maturation is accelerated in the presence of antenatal inflammation[14,25–30]. Increased endogenous cortisol levels have been suggested as the possible mechanism for this relationship, since cortisol can increase surfactant synthesis in the presence of inflammation as well as increase pulmonary fluid clearance and subsequent gas exchange in the presence of MMPs and neutrophils[25].

### Different respiratory morbidity in twin pairs

In twin pairs, the second twin is at increased risk of respiratory morbidity as compared with the first twin[11–13]. Some have suggested that immediate postnatal depression may be attributed to the increased incidence of respiratory complication, but the development of respiratory distress syndrome due to surfactant deficiency is independent of birth asphyxia, gestational age, and mode of delivery[13,31]. Other mechanisms for the observed higher incidence of respiratory morbidity in the second twin have been suggested, including decreased exposure to the protective effects of labor, the development of acute uteroplacental insufficiency after delivery of the first fetus, and lower production of surfactant[9,11].

In this study, we postulated that a relatively lack of exposure to inflammatory mediators may provide the underlying mechanism to explain the differences in lung maturation between twin pairs. To this end, we compared the concentrations of inflammatory cytokines in mid-trimester amniotic fluid between twin pairs. The AF concentrations of IL-8, MMP-8, and CRP were higher in the first twin, and this difference remained significant after adjustment for the fetal growth restriction at the time of birth and chorionicity. We included only twin pregnant women who delivered at term, because intra-amniotic infection and/or inflammation in midtrimester amniotic fluid itself is associated with an increased risk of preterm delivery[32–36].

### Strengths and Limitations

To our knowledge, this is the first study comparing levels of inflammatory cytokines in asymptomatic mid-trimester AF between twin pairs. To avoid the confounder of preterm birth, we limited our analysis only to twin pregnancies who delivered at term. Even in these twin pregnancies, the concentrations of inflammatory markers in mid-trimester AF were higher in the first twin within twin pairs. This could indeed provide a possible explanation for the decreased incidence of respiratory morbidity in the first twin, even in the late preterm or term period, and even after cesarean delivery in the absence of labor[11].

There are several limitations of our study. First, because of a relatively small number of cases and inclusion of only those twin pregnancies that delivered at term, the association between mid-trimester AF concentrations of inflammatory markers and the subsequent development of respiratory morbidity could not be examined in further detail. In the current study population, respiratory morbidity occurred in only one case. In this case, the mid-trimester AF concentrations of IL-8 was 238.8 pg/mL (very low), MMP-8 was 1724.1 pg/mL (low normal), and CRP was 252.2 ng/mL (high) (see Table 2). Further studies are needed to examine the association between mid-trimester exposure to intra-amniotic inflammation and subsequent respiratory morbidity. Second, it is possible that the presenting twin identified in mid-pregnancy may not be the presenting twin at birth. According to one study, a discrepancy between antenatal labeling and the anticipated birth order was observed in about 30% of cases[37]. Third, the study included a measurement of AF cytokines at only a single point in time. Serial AF concentrations of these inflammatory biomarkers would be more useful in helping to understand the relationship between antenatal inflammation and fetal lung development, but this does not seem to be plausible in human studies. Lastly, IL-6 is well known marker for inflammatory condition, and the elevated AF concentrations of IL-6 has been reported to be associated with intra-amniotic infection and adverse pregnancy outcomes [32,38,39]. However in the current study, the mid-trimester AF concentrations of IL-6 were higher in the presenting twin compared with the non-presenting twin, but did not reach statistical significance. Further studies with a larger study population are needed to demonstrate the role of mid-trimester AF IL-6 in the pathogenesis of preterm birth.

In conclusion, as early as the mid-trimester, the presenting fetus in an otherwise uncomplicated twin pregnancy is exposed to higher levels of pro-inflammatory mediators (IL-8, MMP-8, and CRP) than its non-presenting co-twin. Whether this pro-inflammatory milieu reduces the risk of neonatal respiratory morbidity at birth or has other functional implications needs to be further evaluated.
